# Effects of Different Materials on Residual Stress Fields of Blade Damaged by Foreign Objects

**DOI:** 10.3390/ma16103662

**Published:** 2023-05-11

**Authors:** Wangtian Yin, Yongbao Liu, Xing He, Hongsong Li

**Affiliations:** College of Power Engineering, Naval University of Engineering, Wuhan 430033, China

**Keywords:** foreign object damage, numerical simulation, residual stress, constitutive relation

## Abstract

Foreign object damage (FOD) is a common mode of failure in high-speed rotating machinery, such as aircraft engines. Therefore, research on FOD is crucial for ensuring blade integrity. FOD induces residual stress on the surface and within the blade, impacting its fatigue strength and service life. Therefore, this paper utilizes material parameters determined by existing experiments, based on the Johnson–Cook (J-C) constitutive model, to numerically simulate impact damage inflicted on specimens, compare and analyze the residual stress distribution of impact pits, and investigate the influence law of foreign object characteristics on blade residual stress. TC4 titanium alloy, 2A12 aluminum alloy, and Q235 steel were selected as foreign objects, and dynamic numerical simulations of the blade impact process were performed to explore the effects of different types of metal foreign objects. This study analyzes the influence of different materials and foreign objects on the residual stress generated by blade impact through numerical simulation, examining the distribution of residual stress in different directions. The findings indicate that the generated residual stress increases with the density of the materials. Additionally, the geometry of the impact notch is also influenced by the density difference between the impact material and the blade. The distribution of the residual stress field reveals that the maximum residual tensile stress in the blade is related to the density ratio, and the residual tensile stress in the axial and circumferential direction is relatively large. It is important to note that a significant residual tensile stress has a detrimental effect on the fatigue strength.

## 1. Introduction

During operation, gas turbines generate high suction forces that can lead to the ingestion of solid objects [1,2], such as sand, abrasive debris, and falling parts, into the airflow channel. These objects can collide with the compressor blades located at the front of the engine at high velocities, causing damage, such as cracks and spalling, on the blade surface. Such damage can have a significant impact on blade performance. The damage caused by foreign objects is different from notches through low-speed and quasi-static impact. The stress concentration and microstructure deformation caused by foreign object damage (FOD) have an important influence on the fatigue life of the blade [3,4]. Additionally, the tensile residual stress generated in blades by FOD may expedite the propagation rate of fatigue cracks, ultimately leading to a reduction in blade fatigue strength and potential fatigue failure; this significantly hinders the safe operation of gas turbines [5]. Therefore, in blade design and maintenance, the impact of residual stress generated by FOD on blade fatigue strength must be fully considered to maintain the reliability and service life of the engine [6]. This damage will affect the strength and stiffness of the material, thereby reducing its fatigue performance. To accurately simulate these phenomena, factors such as material constitutive relations, yield criteria, failure criteria, etc., must be considered. For gas turbines, the size of the impact object is usually on a millimeter scale, and the residual stress generated by the impact is not easy to measure in practice. Therefore, the use of an FOD numerical simulation method can be useful, as it can effectively describe the size and distribution of stress concentration [7], which is an effective method of studying the FOD damage law.

Chen et al. [8] used the finite element method to study the residual stress produced by spherical indentation. The distribution of the residual stress field was analyzed under different indentations, the influence of residual stress on the stress intensity factor at the crack tip was determined, and the exact approximate analytical solution of the stress intensity factor was given. It was concluded that FOD can reduce the critical crack size by up to 60%. Peters et al. [9] investigated how foreign object damage to a blade affects the initiation and growth of fatigue cracks and calculated the stress intensity factor range for FOD-induced crack initiation and propagation. They suggested that FOD reduces fatigue life mainly by causing microcracks at the impact pits’ edges. Oakley et al. [10] established an elastic crack closure prediction method that considers the stress ratio and residual tensile stress, based on Hutchinson’s calculation of the residual stress generated by FOD, and estimated the stress intensity factor threshold for fatigue crack initiation. Chen et al. [11] indicated that the diameter and depth of craters, as well as their residual stress values, increased with increasing impact velocity of foreign objects. Furthermore, the residual tensile stress near the rim of the craters promoted the initiation of fatigue cracks. Wu et al. [12] showed that the synergistic mechanism resulting from the interactions between micro-damage, micro-structure, and residual stress induced by FOD can increase uncertainty regarding the location of fatigue crack initiation. They evaluated the fatigue crack resistance of FOD axle specimens by considering factors such as fatigue strength degradation, crack closure effect, and the location of crack initiation. Zhao [13] used numerical simulation to analyze the residual stress in the FOD region of the leading edge of a TC17 simulated blade. He calculated the process of a steel ball impacting the leading edge of the simulated blade at different angles, and studied the effect of impact angle on the residual stress distribution at the FOD location. The blade’s fatigue strength decreased due to the tensile residual stress induced by external impacts, whereas uniform compressive residual stress enhanced it. Shot peening is thus utilized in practice to establish a compressive residual stress layer on the blade’s surface, thereby enhancing its fatigue performance. Spanrad et al. [14,15] compared the high-cycle fatigue characteristics of laser shot peening (LSP)-treated and untreated samples subjected to FOD impact under the same load conditions. A delay in crack initiation was observed in the samples, and the results showed that residual compressive stress caused by LSP improves crack growth resistance after FOD. Luo Sihai et al. [16] used finite element software ABAQUS to simulate the influence of LSP and FOD on the blade stress field. The results showed that the residual compressive stress of the LSP-treated blade was greater than that of the untreated blade, thereby reducing the growth rate of cracks. Jia et al. [17] conducted high cycle fatigue tests on annealed specimens without treatment after impact and with residual stress removed after impact, and concluded that the effect of residual stress on fatigue strength is within 10%. Luis [18] characterized the microstructure and residual stress of the material around the crack area using an optical microscope and an X-ray diffractometer. The author mainly studied the residual stress caused by plastic deformation in a notched T-6061 aluminum sample under cyclic load. Using a synchrotron X-ray, Hisao [19] employed the sin^2^ψ technique to measure the residual stress field induced by the impact of a hard sphere on the surface of the Ti-6Al-4V alloy. He found that extensive residual tensile stress existed inside the crater, which would have a detrimental effect on fatigue strength.

This research aims to investigate the effect of foreign object damage on the performance of gas turbine blades using a numerical simulation approach based on the J-C constitutive model. The impact damage inflicted on specimens is simulated numerically, utilizing material parameters obtained from previous experiments. The residual stress distributions resulting from impact pits are analyzed and compared to explore the influence of foreign object characteristics on blade residual stress. This article analyzes the influence of different materials and foreign objects on the residual stress generated by blade impact through numerical simulation, and studies the distribution of residual stress in different directions. Based on these results, the influence of residual stress on blade fatigue strength is discussed.

## 2. Materials and Methods

### 2.1. Material Parameters and Constitutive Model

There are various constitutive models that can be used to describe the mechanical behavior of metal materials under high strain rates, including the Johnson-Cook (J-C) [20], Zerilli–Amstrong (Z-A) [21], Cowper–Symonds (C-S) [22], and Bammann [23] models. These models have different characteristics and can each be suitable for different scenarios. The J-C model is an empirical constitutive model that can comprehensively consider various factors affecting the mechanical behavior of materials, including strain, strain rate, and temperature. It is widely used to predict high-speed deformation and failure behavior and is applicable to isotropic materials. The Z-A model is a constitutive relationship model based on the thermal activation dislocation motion theory, and can reflect the influence of a material lattice’s structure on its mechanical properties. This model can more accurately describe the strain rate effect of materials. The C-S model considers the effect of strain rate in a relatively simple form and is widely used in impact finite element simulations. Although its applicability is relatively narrow, it can rapidly provide simulation results. The Bammann model is a viscoplastic constitutive model based on internal state variable theory. This model can consider the effects of dislocation hardening, void damage, plastic anisotropy, etc., under high-speed impact conditions, and can effectively describe the viscoplastic deformation characteristics of materials. In summary, different types of material have different degrees of response to strain rate sensitivity. Choosing an appropriate model based on the application scenario and requirements can result in a better description of the mechanical behavior of materials under high strain rates. Different materials have different responses to strain rate sensitivity. Therefore, it is important to choose appropriate models based on the loading conditions and material properties. These models require calibration and verification using experimental data, as well as consideration of their applicability and limitations. Different materials also display distinct behavior and failure mechanisms under high strain rates. This highlights the need to carefully select the appropriate type and establish the corresponding constitutive relationship for specific circumstances.

The J-C model is widely used in engineering and research because the product relationship among its various variables is simple and its parameters are relatively easy to obtain. However, as an empirical model, the parameters of the model are obtained from multiple material experiments, making them non-universal across different materials. For high-speed impact events, the combination of the J-C model and its corresponding dynamic damage model can more accurately reflect the mechanical response of metal materials. Therefore, the J-C constitutive model is also used to study the blade’s response to foreign objects.

The material strength of the J-C model depends on plastic strain, strain rate, and temperature [24], so the model can be written as Equation (1):

(1) $$\sigma_{eq}=\left(A+B\varepsilon_{eq}^{n}\right)\left(1+C\ln{\dot{\varepsilon }}_{eq}^{*}\right)\left(1-T^{*m}\right)$$

where ***σ****_eq_* denotes the equivalent stress (MPa), ***ε****_eq_* is the equivalent plastic strain, ${\dot{\varepsilon }}_{eq}^{*}$ is the dimensionless equivalent plastic strain rate defined as the ratio of the equivalent plastic strain rate, ${\dot{\varepsilon }}_{eq}^{}$ (s^−1^),to the reference strain rate, ${\dot{\varepsilon }}_{0}^{}$ (s^−1^), the parameters *A*, *B*, *C*, *m*, and *n* are material constants, and T* is the normalized temperature (K), defined as

(2) $$T^{*}=\frac{T-T_{room}}{T_{melt}-T_{room}}$$

where *T* is the material temperature (K), *T_melt_* is the melting temperature (K), and *T_room_* is the room temperature (K).

According to the cumulative damage law of the J-C material model, damage can be expressed as Equation (3):

(3) $$D=\sum \frac{\Delta \varepsilon_{eq}}{\varepsilon_{f}}$$

where *D* is the damage parameter, Δ*ε_eq_* is the increment of effective plastic strain, and *ε_f_* is the failure strain. The failure strain can be written as Equation (4)

(4) $$\varepsilon_{f}=\left(D_{1}+D_{2}e^{D_{3}\cdot \sigma^{*}}\right)\left(1+D_{4}\ln{\dot{\varepsilon }}_{eq}^{*}\right)\left(1+D_{5}T^{*}\right)$$

where $\sigma^{*}=-p/\bar{\sigma }$ is the stress triaxiality defined as the ratio of the pressure, *p* (MPa), to the von Mises stress, $\bar{\sigma }$ (MPa), and *D*_1_–*D*_5_ are the material parameters (see Table 1).

This paper endeavors to advance the field of foreign object damage (FOD) testing by utilizing existing publications that report on the properties of various materials. Through a comprehensive analysis and comparison of blade performance under varying material impact conditions, this paper seeks to deepen our understanding of this critical research area. The cited publications serve as a valuable source of data for this analysis. Wei, Lin et al. [25,26,27,28]. carried out detailed studies of the material properties of 2A12 aluminum alloy, Q235 steel and TC4 titanium alloy, and calculated the relevant parameters of the J-C constitutive model and fracture criterion. In this study, the material parameters of the three metals were utilized to simulate the stress state of the damaged blades under the influence of strain and strain rate. These parameters are listed in Table 1.

### 2.2. Dynamic Numerical Simulation

**Figure 1:**
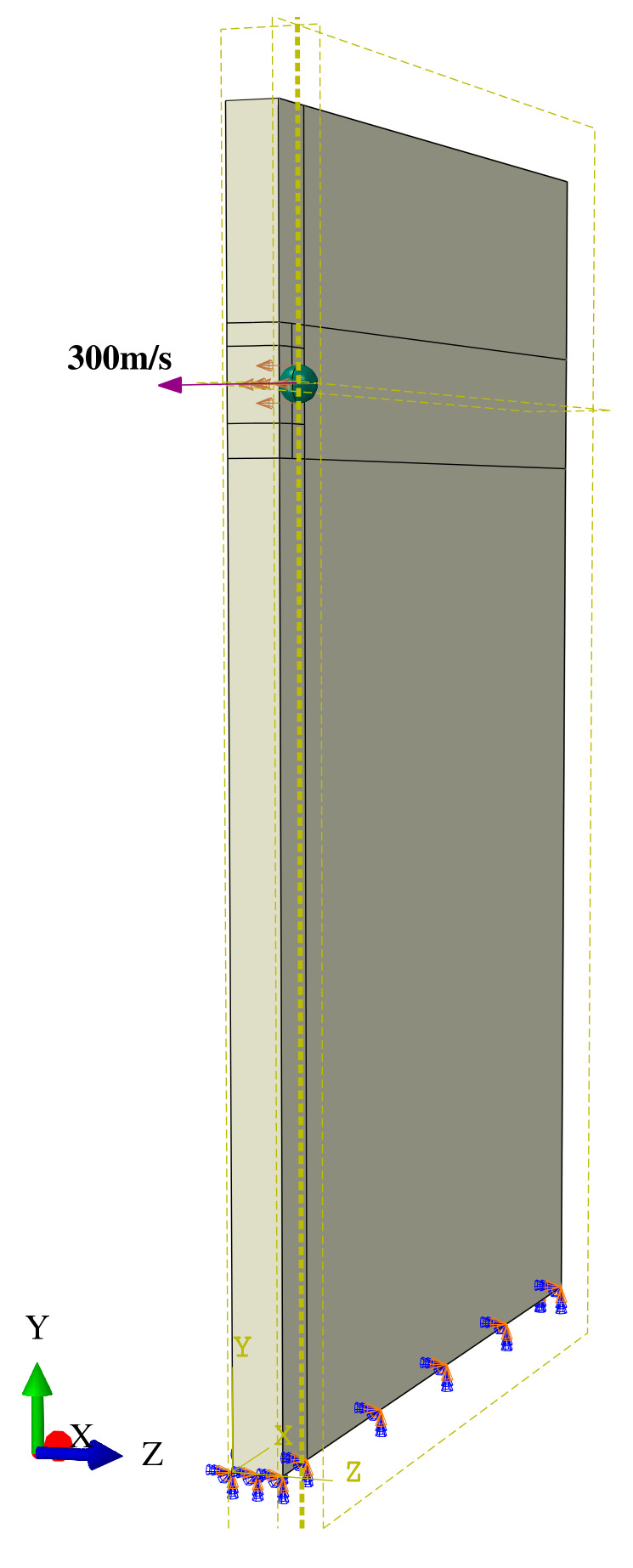
Diagram showing 80% of blade height impacted by foreign objects.

In this study, ABAQUS 6.14-4 finite element software was employed to simulate the damage process of foreign object impact on a gas turbine compressor blade. The use of explicit expressions allowed for a more accurate analysis of instantaneous loads as well as precise calculation of the structural response. To ensure the fidelity of the simulation, the model was defined as follows:

(1) This paper investigates the FOD behavior of the TC4 titanium alloy, a widely used material for aero-engine compressor blades;
(2) In order to simulate the gas turbine blade, a rectangular specimen with a size of 75 mm × 56 mm × 3 mm was used as a model of the geometric shape of the blades to simplify the simulation. This simplification made it possible to focus on the mechanical response characteristics of the impact without considering the complex curvature of the blade;
(3) The position where foreign objects collide with a blade is generally between at 60% and 100% of the blade height, so 80% of the blade height was selected as the impact point [29]. A hard spherical object with a radius of 1.5 mm was used to execute the impact at 80% of the blade height, on its leading edge, at a speed of 300 m/s, as shown in Figure 1.

To accurately simulate the blade impact process, displacement constraints were added to the clamping part of the blade model, and the surface-to-surface contact collision type was selected. The Advancing Front Algorithm was utilized to control the mesh, the C3D8R mesh type was chosen for both foreign objects and blades, and the hourglass control method was applied for enhanced control. By refining the mesh in the impact area, the calculation accuracy was further improved. Overall, the established finite element model provided a reliable basis for simulating the FOD process of gas turbine compressor blades and analyzing the resulting damage.

**Figure 2:**
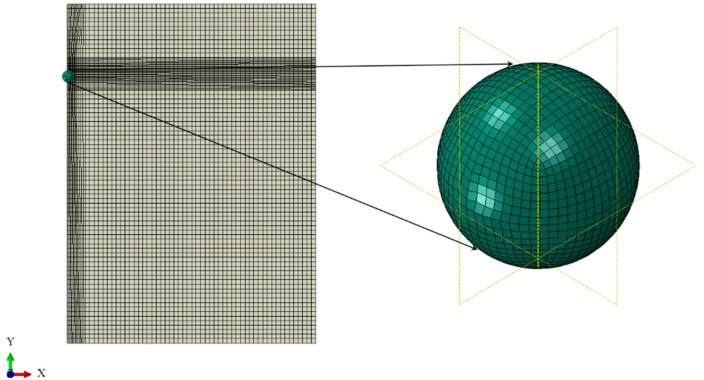
Local mesh densification.

In ABAQUS analysis, it is necessary to verify the grid independence of the numerical simulation results. Due to the time difference, the time increment step is small enough to obtain accurate results. The meshed model used in this study is depicted in Figure 2. On the right side of the image is a partially enlarged view of the foreign object grid. This enabled the simulation to capture the behavior of the blade during the impact process and produce more accurate results.

### 2.3. Verification of Constitutive Model

**Figure 3:**
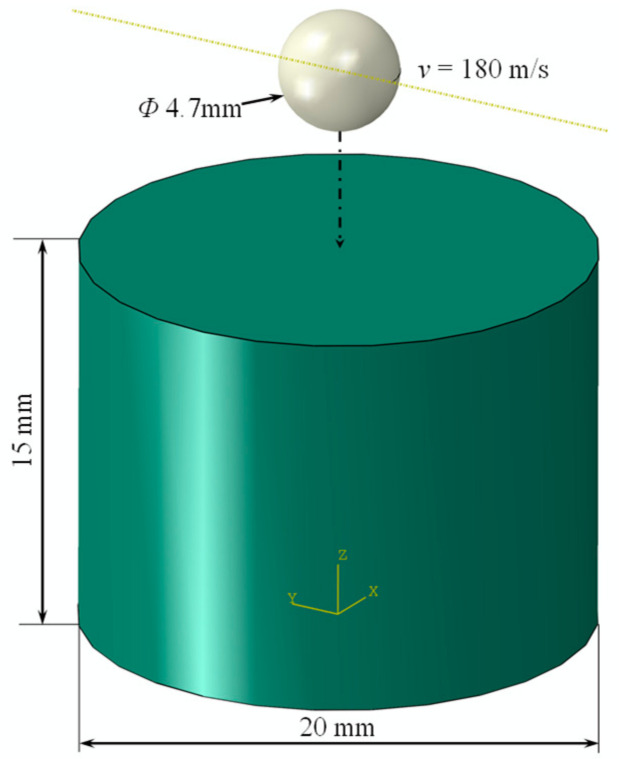
Conditions for object impact specimen.

To verify the accuracy of the J-C constitutive model in ABAQUS, experimental results from reference [19] were utilized. The experiment involved the impact of a 4.7 mm diameter steel ball at a speed of 180 m/s on the center of the specimen. Figure 3 illustrates a schematic diagram of the impacted specimen.

**Figure 4:**
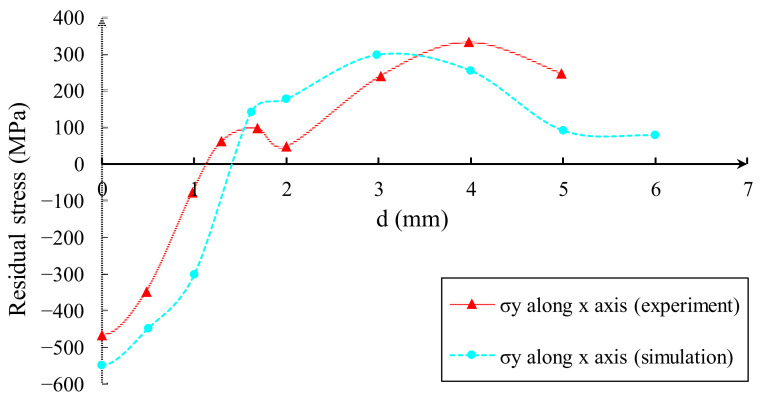
Distributions of residual stress for experiment and simulation (data taken from [19]).

By comparing the numerical simulation results using the J-C model with the experimental data, it was observed that the model is symmetric about the *Z*-axis, allowing for the calculation of stress in a single direction to verify the results. Figure 4 shows that, at the center of the crater, the compressive stress values were −470 MPa for the experiment and −547 MPa for the simulation, resulting in an error of 16.4%. As the maximum allowable error between experimental and simulated values in the entire residual stress field distribution is 20%, it can be concluded that the numerical simulation model employed in this study is capable of accurately simulating actual results.

## 3. Results

### Residual Stress Distribution and Analysis

A dynamic impact simulation of TC4 titanium alloy blades impacted with different types of metals was carried out, and the sizes of the pits and sthe size of the residual stress in all directions were compared.

**Figure 5:**
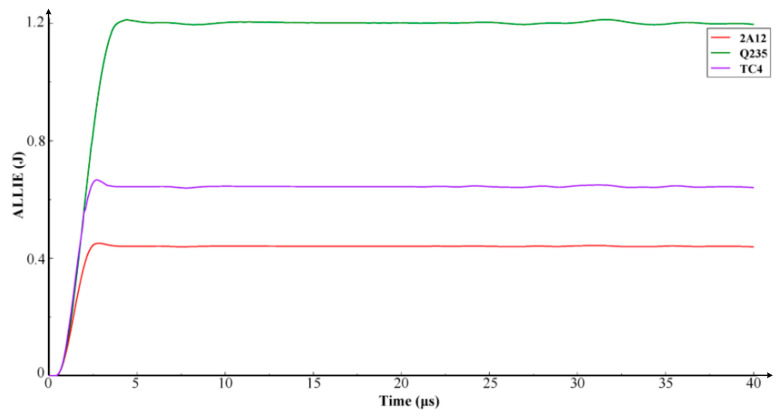
The change in the internal energy of the blades impacted by different materials at a speed of 300 m/s.

Figure 5 shows how the total internal energy of the system varies with the material density of the ball and the impact energy, which is due to density and velocity. It also shows that the internal energy of the system stabilizes in 5 × 10^−6^ s, and the stress distribution after this is represented by a stable residual stress field.

**Figure 6:**
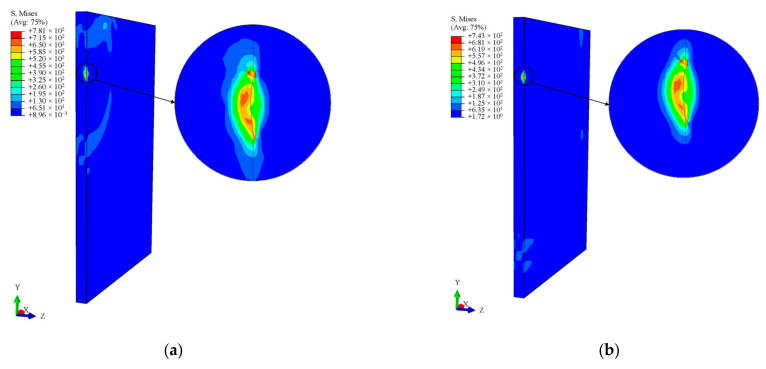

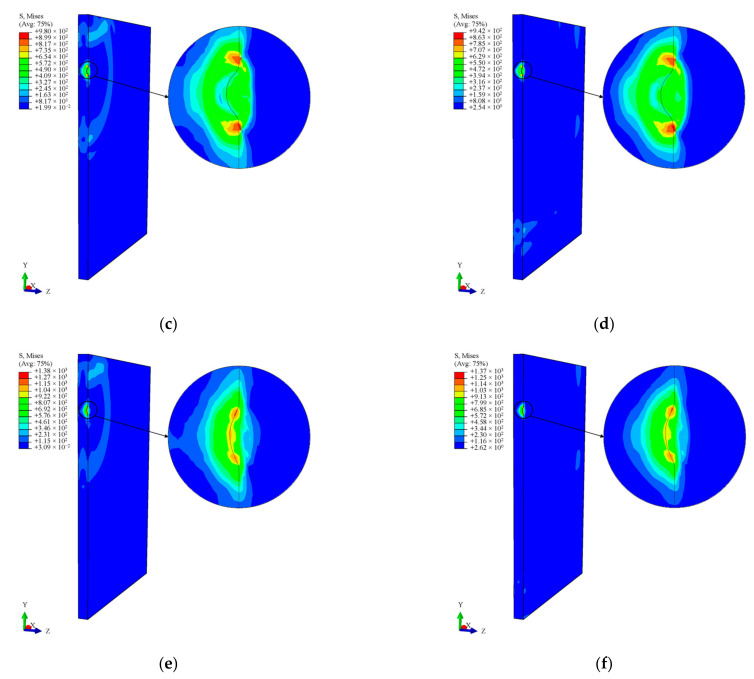
Cloud diagram of blade stress distribution at different times. (**a**) Equivalent residual stress Cloud diagram of aluminum impact blade at 1 × 10^−5^ s. (**b**) Equivalent residual stress Cloud diagram of aluminum impact blade at 3 × 10^−5^ s. (**c**) Equivalent residual stress Cloud diagram of TC4 impact blade at 1 × 10^−5^ s. (**d**) Equivalent residual stress Cloud diagram of TC4 impact blade at 3 × 10^−5^ s. (**e**) Equivalent residual stress Cloud diagram of Q235 impact blade at 1 × 10^−5^ s. (**f**) Equivalent residual stress Cloud diagram of Q235 impact blade at 3 × 10^−5^ s.

The internal energy of the system increases differently when impacted by foreign objects of the same size but composed of different materials. This is due to differences in the material density and elastic modulus of the objects. The higher the elastic modulus of the material, the more internal energy it adds to the system. Figure 6 shows how stress is distributed in the 2A12 aluminum alloy, TC4 titanium alloy, and Q235 steel when a spherical hard object hits the blade. Figure 6a–f represent snapshots of stress at different times. The stress wave spreads from the impact crater to the surrounding area in 1 × 10^−5^ s. The stress state of the flat plate specimen stabilizes in 3 × 10^−5^ s, and the residual stress field distribution is obtained. The maximum residual stress at the impact point is 60% of its value during impact, and its distribution range is wider. The peak value of residual stress also depends on the density and elastic modulus of the impactor. Q235 steel has the highest residual stress at the impact point, followed by TC4 titanium alloy and 2A12 aluminum alloy, the latter of which has the lowest residual stress.

## 4. Discussion

A dynamic impact simulation of TC4 titanium alloy blades impacted with different types of metal was carried out, and the sizes of residual stress caused by each material were compared. Due to the differences in material density, the impact of foreign objects of the same size increased the internal energy of the system. This increase is positively correlated with the density of the material.

**Figure 7:**
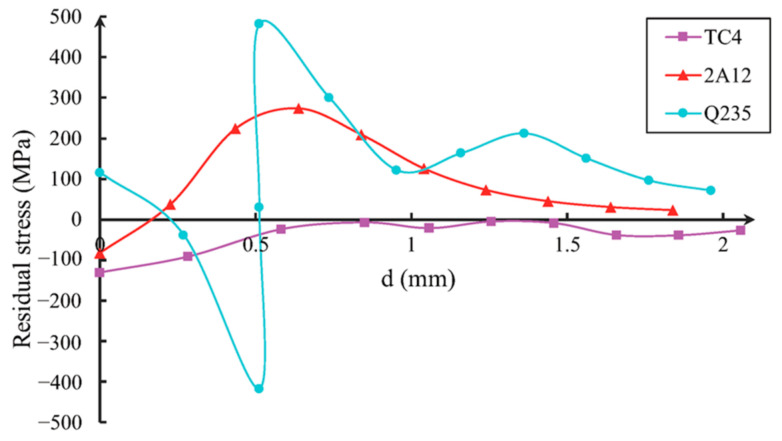
Residual stress σ_X_ distribution along axis direction.

**Figure 8:**
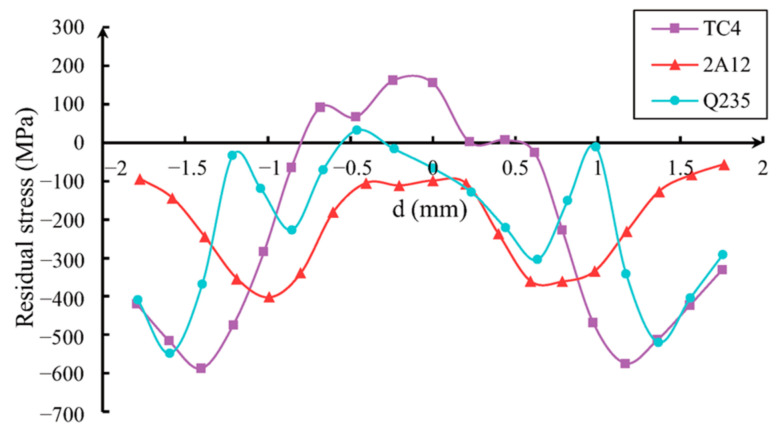
Residual stress σ_Y_ distribution along radial direction.

**Figure 9:**
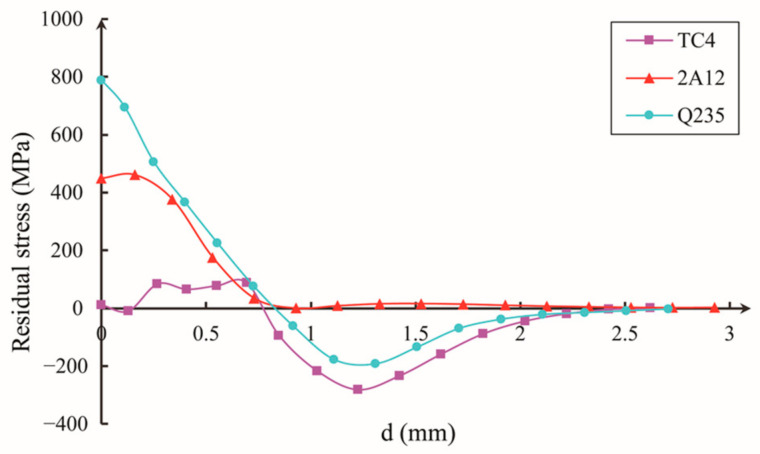
Residual stress σ_Z_ distribution along circumferential direction.

The numerical results of the residual stress generated by different material models at a speed of 300 m/s were compared. Figure 7, Figure 8 and Figure 9 show the residual stress distribution curves in the X-direction (axial direction), Y-direction (radial direction), and Z-direction (circumferential direction) near the impact crater. The abscissa d in the figure represent the distance from the specified starting position, and the starting position is at the center of the crater.

The integrity of gas turbine blades is largely determined by their residual tensile stress component. Figure 7, Figure 8 and Figure 9 illustrate that the residual stress resulting from projectile impacts is particularly pronounced in the axial and circumferential direction.

It can be seen from Figure 7, Figure 8 and Figure 9 that, along the same direction, different materials cause different residual stress changes after impact. Along different directions, changes in the residual stress caused by the same material after impact are also different.

At the impact point, the residual stress from the Q235 steel ball in the axis direction (along the *X* axis) changes from a tensile state to a compression state. Due to the hardness of the ball compared to the test piece, there are visible annular protrusions in the pits generated at the edge of the test piece, so the residual stress along the axis direction undergoes a sudden change at this position. The density of the 2A12 aluminum ball is lower than that of the test piece, so the ball deforms significantly, and the residual stress component at the pit position of the test piece becomes compressed. However, due to the extrusion effect of the material, tensile stress occurs at the annular protrusion where the pit occurs, but the extreme value of the residual stress is smaller than the extreme value of the residual stress from Q235. At the same time, it can be seen that the residual stress generated by TC4 impact is between the other two materials, and in the two directions with higher residual tensile stress (axial and circumferential direction), the residual tensile stress value of TC4 is smaller than that of the other two materials. This indicates that residual stress is related to the density ratio of the impacted object to the blade.

Based on Figure 9, it can be observed that the maximum value of the residual tensile stress component in the circumferential direction (σ_Z_) is located at the center of the notch. The maximum residual tensile stress values in this region are −800 MPa for Q235 and −461 MPa for 2A12. The yield strength of TC4 alloy is 860 MPa, so the residual tensile stress in the *Z*-axis direction caused by the impact of Q235 material is close to the yield stress of the blade, reaching 93% of the yield stress. Therefore, the residual tensile stress in FOD cannot be ignored.

## 5. Conclusions

This study utilized the J-C model and the finite element method to assess the impact damage caused by spherical metal on compressor blades. The distribution of residual stress was compared and analyzed, leading us to draw the following conclusions:

(1) When hard objects of the same size but composed of different materials impact the blade at the same speed, the size of the residual stress generated varies and is positively correlated with the density of the material. At the same time, the geometric size of the impact crater is related to the density of the material and the maximum residual tensile stress of the blade is related to the density ratio.
(2) The residual stress caused by the impact of hard objects cannot be ignored, and the influence of residual stress should be considered when calculating the high-cycle fatigue life of damaged blades. The maximum residual tensile stress in the *Z*-axis direction caused by Q235 material impact reaches 93% of the blade yield stress.
(3) The residual stress field generated by FOD has different trends in different directions, among which the residual tensile stress in the axial and circumferential direction is relatively large, and the reduction effect on the fatigue strength of the blade needs special attention.
(4) Based on the above simulation results, we suggest that the evaluation of fatigue strength degradation caused by FOD must consider not only geometric changes, but also the type of material and the relative density of the specimen.

## Figures and Tables

**Table 1:** Material parameters (data taken from [25,26,27,28]).

Materials		Parameters											
	ρ/(kg/m^3^)	E/GPa	υ	*A*	*B*	*n*	*C*	*m*	*D*_1_	*D*_2_	*D*_3_	*D*_4_	*D*_5_
TC4	4430	105	0.31	1078	1092	0.38	0.014	1.1	−0.09	0.27	−0.48	−0.014	3.87
2A12	2770	71.7	0.33	400	989	0.654	0.001	1.426	0.116	0.211	−2.172	0.012	−0.01256
Q235	7850	200	0.33	244.8	899.7	0.94	0.0391	0.757	−43.408	44.608	0.016	0.0145	0.046

## Data Availability

Not applicable.
